# Shear Performance Degradation of Fiber-Reinforced Recycled Aggregate Concrete Beams Under Salt Freeze–Thaw Cycles

**DOI:** 10.3390/ma18204817

**Published:** 2025-10-21

**Authors:** Shefeng Guo, Jin Wu, Jingmiao Zhao, Zhehong Zeng, Xiangyu Wang, Yiyuan Wang, Haoxiang Luan, Yulin Wang, Dongxia Hu

**Affiliations:** 1 College of Civil Aviation, Nanjing University of Aeronautics and Astronautics, Nanjing 211106, China; bt1601009@nuaa.edu.cn (S.G.); zjm07282000@163.com (J.Z.); zzh18205901450@163.com (Z.Z.); 15161768550@163.com (X.W.); 13919148253@163.com (Y.W.); luanhaoxiang@nuaa.edu.cn (H.L.); 15951671180@163.com (Y.W.); hudongxia_xj@163.com (D.H.); 2 Xinjiang Vocational & Technical College of Communications, Urumqi 831401, China

**Keywords:** composite salt freeze-thaw, polypropylene fiber, recycled aggregate concrete beam, shear performance degradation

## Abstract

In saline soil and alpine regions of northwest China, fiber-reinforced recycled aggregate concrete (FR-RAC) beams are subjected to coupled degradation from a chloride–sulfate composite salt attack and freeze–thaw cycling. Existing studies predominantly focus on natural aggregate concrete in freshwater environments or single-salt solutions, with limited documentation on the shear performance of FR-RAC beams after freeze–thaw exposure in chloride–sulfate composite salt solutions. To investigate the durability degradation patterns of FR-RAC beams in Xinjiang’s saline soil regions, two exposure environments (pure water and 5% NaCl + 2.0% Na_2_SO_4_ composite salt solution) were established. Shear performance tests were conducted on nine groups of FR-RAC beams after 0–175 freeze–thaw cycles, with measurements focusing on failure modes, cracking loads, and ultimate shear capacities. The results revealed that under composite salt freeze–thaw conditions: after 100 cycles, the cracking load and shear capacity of tested beams decreased by 39.8% and 22.2%, respectively, compared to unfrozen specimens representing reductions 29.6% and 82.0% greater than those in freshwater environments; at 175 cycles, cumulative damage intensified, with total reductions reaching 56.8% (cracking load) and 36.1% (shear capacity). A shear capacity degradation prediction model for FR-RAC beams under composite salt freeze–thaw coupling was developed, accounting for concrete strength attenuation and interfacial bond degradation. Model validation demonstrated excellent agreement between predicted and experimental values, confirming its robust applicability.

## 1. Introduction

Recycled Aggregate Concrete (RAC) represents a core technology for converting construction waste into resources, with significant sustainability contributions to global environmental and resource security. According to the United Nations Environment Programme (UNEP, 2023) [1] in its Global Status Report for Buildings and Construction 2023, the building sector (including building operations and material production) accounts for 37% of global energy-related CO_2_ emissions, with a notable proportion stemming from the production of building materials. Additionally, the over-exploitation of natural aggregates has exacerbated pressure on resource consumption. RAC, by transforming construction waste into recycled aggregates, effectively reduces reliance on the extraction of natural aggregates and lowers carbon emissions during concrete production (by minimizing energy consumption associated with natural aggregate extraction, transportation, and construction waste landfilling). This provides crucial technological support for the construction industry to achieve its carbon neutrality and net-zero emission goals. By reprocessing demolished concrete waste into recycled aggregates for concrete production, RAC addresses both the resource depletion caused by over-exploitation of natural aggregates and the environmental pressure from construction solid waste landfills. This technology holds irreplaceable strategic value for advancing low-carbon transformation in civil engineering [2,3]. However, recycled aggregates have an inherent characteristic—surface-attached old mortar—which creates structural weaknesses in the interfacial transition zone (ITZ), leading to low interfacial bond strength and high initial porosity [4]. This further causes mechanical performance degradation: with the same mix proportion, RAC’s compressive strength is 10–40% lower than that of natural aggregate concrete (NAC), and durability indicators (e.g., frost resistance, impermeability) also decrease significantly [5]. This limitation directly restricts the large-scale application of RAC in load-bearing structures like bridges and roads.

From the perspective of engineering practice, concrete structures in mid-to-high-latitude regions (e.g., northwest China, northern Europe) face prolonged freeze–thaw cycles. The expansion of pore water during freezing accelerates concrete cracking [6,7,8]. Due to its higher initial porosity, RAC experiences faster frost damage than NAC [9]. More critically, RAC structures in high-altitude saline-soil-regions like northwest China must simultaneously withstand combined NaCl-Na_2_SO_4_ salt attack. Research has demonstrated that the synergistic effect of salt ions and freeze–thaw cycles can exacerbate damage. The underlying mechanism lies in the fact that the chemical reactions of sulfate ions induce cracking in the interfacial transition zone, while freeze–thaw cycles accelerate the penetration of salt ions. Together, these two factors create a vicious cycle of “physical damage—chemical erosion” [10,11].

It is noteworthy that in shear-dominated structural members (such as beam–column joints), the residual mortar adhering to the surface of recycled aggregates further exacerbates the performance degradation of the ITZ. Compared to NAC, the ITZ between recycled aggregates and new mortar in RAC exhibits higher porosity and lower bond strength, making it prone to becoming stress concentration points under shear loading and accelerating the initiation and propagation of diagonal cracks (Etxeberria et al., 2007; Pradhan et al., 2018) [12,13]. Moreover, salt freeze–thaw cycles further weaken the bond performance of the ITZ, resulting in a significantly greater reduction in shear capacity for RAC shear members compared to NAC members (Fathifazl et al., 2011) [14]. Therefore, clarifying the mechanical performance evolution of RAC structures under combined salt freeze–thaw environments has become a critical prerequisite for promoting RAC applications in cold regions.

Current research on “mechanical performance of concrete beams under freeze-thaw environments” has focused on NAC beams with notable limitations in research scope and scenarios. For NAC beams, mature understanding has been established: Cao et al. [15] found through 0–150 freeze–thaw tests that cracking load and ultimate shear capacity of NAC beams decrease linearly with freeze–thaw cycles; Duan et al. [16] confirmed that frost action primarily reduces beam ductility by weakening concrete matrix strength while minimally affecting crack distribution patterns. Building on this, Wei [17], Berto et al. [18], and others developed bending and shear capacity prediction models for post-freeze–thaw NAC beams, showing good agreement with experimental data (errors ≤ 10%). These studies provide foundational insights into freeze–thaw mechanical behavior but fail to extend to RAC due to significant ITZ structural differences; NAC features dense interfaces, while RAC exhibits porous ITZs with old mortar residues.

Research on RAC beam shear performance remains concentrated on normal-temperature, salt-free conditions [19,20] without reaching consensus: on one hand, most studies indicate that RAC beams exhibit decreasing shear strength with increasing RCA replacement ratio [21,22,23]. For example, Rahal (2017) [22] found that beams with 10–100% RCA had a 13–18% lower shear strength than NAC beams, and Setkit (2021) [21] confirmed that 100% RCA beams had a 6% lower normalized shear stress, with over 80% of specimens showing strength reduction when the replacement ratio was ≥50%. Pradhan et al. (2018, India) [13] further clarified the shear performance characteristics of RAC beams through comparative tests on 14 beams (7 RAC beams and 7 NAC beams). Even when the particle packing method was employed to optimize the concrete mix proportion, the ultimate shear strength of RAC beams without stirrups was still 14% lower than that of NAC beams under the same conditions. Fathifazl et al. (2011, Canada) [14] pointed out in their study that the residual mortar adhering to the surface of RCA leads to the formation of weak interfacial zones within RAC, which are prone to interfacial failure under shear action. Aggregate interlocking is one of the key mechanisms for the shear resistance of concrete beams, and interfacial failure directly undermines this effect. Moreover, it accelerates the initiation and propagation of diagonal shear cracks within the beam, causing the shear failure process of RAC beams to progress faster than that of NAC beams under the same conditions. Hossain (2023) [23] observed similar trends in composite systems, with 50% RCA beams showing 8–13% shear strength reduction despite fiber modification. On the other hand, design codes like ACI 318 and Eurocode 2 conservatively apply NAC shear provisions to RAC beams but face limitations: (1) the predictions of existing codes for the shear strength of recycled concrete beams tend to be conservative. For instance, the calculated values according to the ACI 318 code are 7–12% lower than the measured values (Rahal, 2017) [22]. Therefore, a correction factor of 0.75 needs to be introduced when applying ACI 318-19 (Setkit, 2021) [21]. Additionally, the study by Etxeberria et al. (2007, Spain) [12] also found that when using the Eurocode 2 code to predict the shear strength of beams with a 50% replacement rate of recycled coarse aggregate, the calculated results also showed deviations. The main reason is that this code fails to take into account the adverse impact of ITZ performance degradation in RAC on aggregate interlocking, and thus cannot accurately reflect the actual shear behavior of RAC beams. (2) Codes fail to address high replacement ratios and severe environments. Zhang (2008) [19] reported 18–23% shear strength reduction in 100% RCA beams, for which codes provide no modification clauses. Wu and Su (2019) [9] confirmed 30–40% faster bearing capacity degradation in RAC beams compared to NAC under freeze–thaw, with codes ignoring coupled damage effects.

To improve RAC performance, researchers have explored mineral admixtures and fiber reinforcement: mineral admixtures like silica fume and fly ash enhance ITZ density through pozzolanic reactions, increasing RAC compressive strength by 15–25% [24,25]. Polypropylene and basalt fibers suppress crack propagation through microcrack bridging, improving shear capacity by 23.44–64.48% [26,27]. However, these modifications are limited to normal-temperature dry environments, and fail to consider coupled freeze–thaw salt effects, providing no support for cold-region RAC beam design.

Critical research gaps persist: (1) insufficient scenario coverage—existing RAC beam shear studies focus on single-freeze–thaw environments without systematic analysis of Cl^−^/SO_4_^2−^–freeze–thaw synergy, which characterizes northwest cold-region engineering conditions; (2) high replacement ratio deficiency—while 100% RCA maximizes construction waste utilization (aligning with “Dual Carbon” goals), its high old mortar content and performance variability lead to avoidance in most studies, resulting in lack of quantitative data on cracking load and ultimate capacity degradation under combined salt freeze–thaw, and absence of corresponding decay models for engineering design.

Given these gaps, this study focuses on the shear performance of 100% RCA RAC beams under combined salt freeze–thaw environments through two main tasks: (1) designing two freeze–thaw scenarios (water and NaCl-Na_2_SO_4_ composite salt simulating northwest saline soil conditions) to conduct 0–175 freeze–thaw tests, observing failure modes (crack distribution, damage patterns) and clarifying salt type impacts on damage mechanisms; (2) quantifying the degradation laws of cracking load and ultimate shear capacity with freeze–thaw cycles based on experimental data, and establish a shear capacity prediction model for RAC beams under combined salt freeze–thaw environments by integrating concrete axial compressive strength decay models, providing a basis for design and safety assessment of cold-region RAC structures.

## 2. Experimental Procedure

### 2.1. Materials and Mixture Proportions

#### 2.1.1. Materials

**Figure 1:**
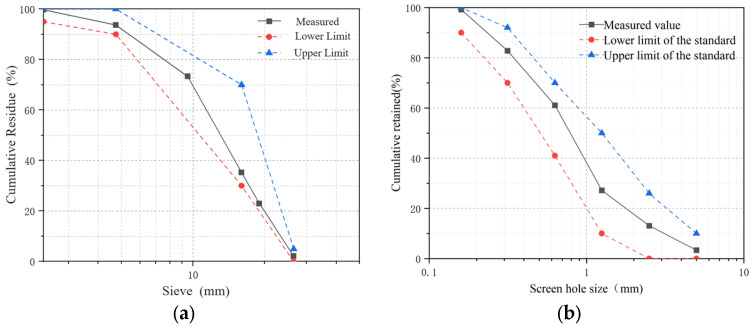
Gradation curves of (**a**) RCA and (**b**) natural fine aggregate (medium sand). (**a**) Schematic diagram of gradation distribution curve for RCA. (**b**) Schematic diagram of gradation distribution curve for medium sand.

The cement used was locally sourced P.O 42.5 Ordinary Portland Cement (Conch brand, produced by China Cement Plant Co., Ltd., Jiangsu, China). All properties comply with the Chinese National Standard Common Portland Cement (GB 175-2023) [28]. Recycled coarse aggregate (RCA) and fine aggregate were processed by Nanjing Shoujia Renewable Resources Utilization Co., Ltd. The test results indicate that RCA consists of continuously graded (5–26.5 mm) crushed stone, with gradation curves shown in Figure 1a. RCA properties include the following: apparent density is 2356 kg/m^3^, crushing value is 16.1%, water absorption is 4.5%, Los Angeles abrasion value is 25%. Fine aggregate properties are as follows: fineness modulus is 2.68, apparent density is 2550 kg/m^3^, bulk density is 1516 kg/m^3^, and clay content is 1.2% (sieve analysis in Figure 1b).

The basic properties of the material are shown in Table 1.

The relevant raw materials used in this study include the following: Class I fly ash (Fly ash, from Henan Xinxiang Huadian Group) and P·O 42.5 grade cement (from Jiangsu Branch of China Cement Plant Co., Ltd.). The key characteristic parameters (D10, D50, D90) of their particle size distributions are presented in Table 2, and the corresponding particle size distribution curves are shown in the relevant figures.

**Figure 2:**
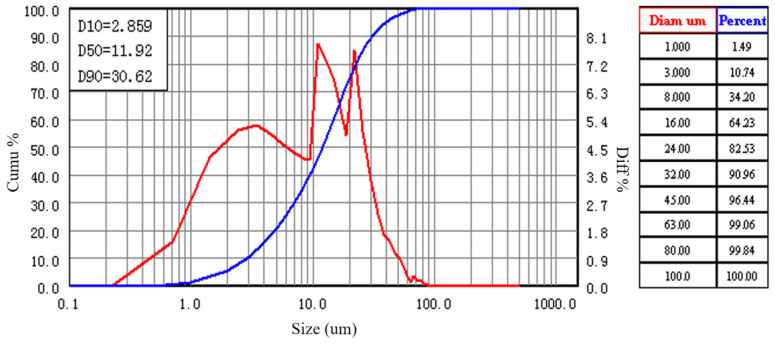
Particle size distribution curve of fly ash (fly ash).

**Figure 3:**
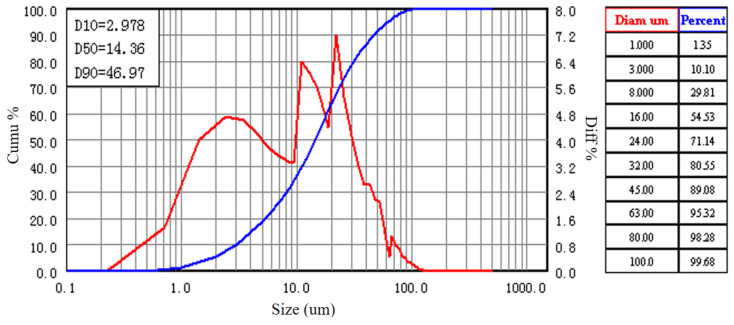
Particle size distribution curve of cement (cement).

From Table 2, it can be observed that the particle sizes of fly ash and cement exhibit a favorable grading relationship. The median particle size of fly ash (D50 = 11.92 μm) is slightly smaller than that of cement (D50 = 14.36 μm). This particle size difference is conducive to a denser particle packing. The spherical shape of fly ash can produce a “ball-bearing” effect, improving workability, while its fine particles can fill the voids between cement particles. The particle size distribution curves of fly ash and cement are shown in Figure 2 and Figure 3.

#### 2.1.2. Mixture Proportions

To enhance the relevance and engineering applicability of this study, the research focused on recycled concrete beams with 100% replacement of recycled coarse aggregate (RCA), specifically targeting freeze–thaw environments in the Lop Nur saline soil region of Xinjiang. The experimental design strictly adhered to the Xinjiang local standard Technical Specification for Recycled Aggregate Concrete (XJJ 076-2017) [30], while incorporating methodologies from prior studies by Wang [20], Huang [31], and Wang [32]. The optimized mix design comprised 100% RCA, 20% fly ash, and 0.9 kg/m^3^ polypropylene fibers. Fly ash improved concrete compactness through filler effects, while polypropylene fibers suppressed microcrack propagation via crack bridging mechanisms, synergistically enhancing freeze–thaw resistance. This mix design meets the technical requirements for C40 freeze-resistant concrete in saline soil environments, as detailed in Table 3.

All the RCA used in this study were obtained from discarded concrete members (mainly beams and columns of frame structures) from local existing building demolition projects. The performance parameters of the parent concrete strictly meet the requirements of the Code for Design of Concrete Structures (GB 50010-2010) [33], with the following specific information.

Parent Concrete Strength Grade: the designed strength grade was C35. Laboratory tests show that the measured 28-day cube compressive strength values range from 36.8 to 38.5 MPa (meeting the code requirements). The RCA produced from members of this strength grade has a measured crushing index of 17.2% and a measured apparent density of 2510 kg/m^3^, both reaching the technical standards for “Class II recycled coarse aggregate” in the RCA for Recycled Concrete (GB/T 25177-2010) [34].

Composition and Proportion of the Parent Concrete: P·O 42.5 ordinary Portland cement was used as the cementitious material. The coarse aggregate was locally sourced limestone crushed stone with a maximum particle size of 25 mm. The fine aggregate was natural river sand with a fineness modulus of 2.6 (classified as medium sand). No mineral admixtures were added in the mix proportion design. The water–cement ratio was 0.45, ordinary drinking water was used as the mixing water, and no concrete admixtures were added.

### 2.2. Specimen Design and Fabrication

#### 2.2.1. Specimen Parameters

**Figure 4:**
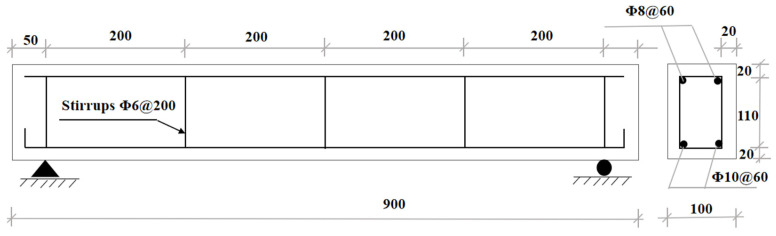
Dimensions and reinforcement details of specimens (mm).

A total of 54 specimens were fabricated, comprising 9 groups of test beams and their associated standard test blocks (including 100 × 100 × 400 mm prisms and 100 × 100 × 100 mm cubes). The test beams were designed as simply supported rectangular sections with dimensions of 100 × 150 mm in cross section, 900 mm in total length, 800 mm in the span, and 20 mm concrete cover thickness. Figure 4 illustrates the beam dimensions and reinforcement configuration.

#### 2.2.2. Specimen Fabrication

**Figure 5:**
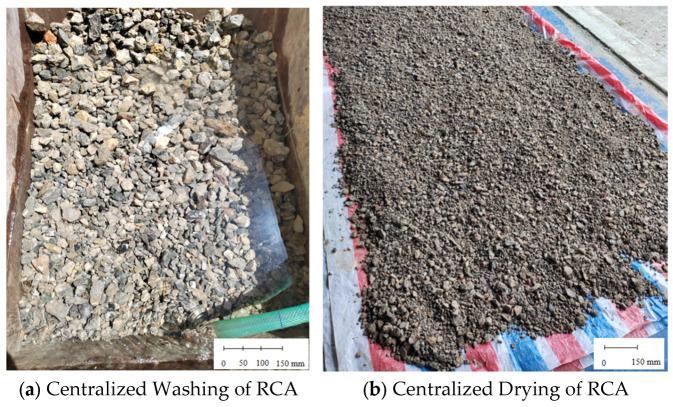
Washing treatment of RCA prior to concrete casting.

To address material quality variability caused by unstable sourcing of recycled coarse aggregates (RCA), all RCA required for this study were procured in a single batch from a local supplier (Nanjing Shoujia Recycling Co., Ltd.) to ensure consistent material properties. Additionally, to mitigate quality risks associated with sediment adhering to RCA surfaces, the aggregates underwent centralized fresh-water washing prior to concrete casting, minimizing sediment content (see Figure 5). All test specimens were fabricated through single-batch centralized casting to eliminate quality variations arising from non-simultaneous casting processes.

**Figure 6:**
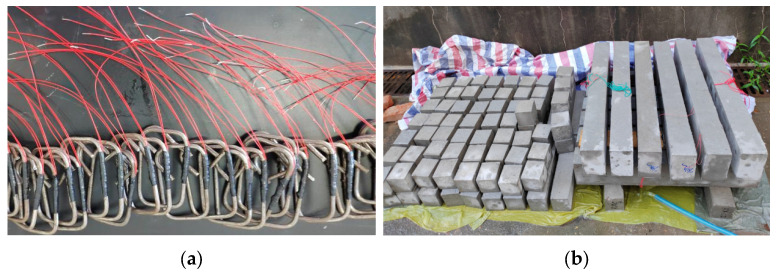
Fabrication of the stirrups and the curing of specimens. (**a**) Strain gauge installation and encapsulation on stirrups. (**b**) Curing for recycled aggregate concrete specimens.

Concrete casting was performed in strict accordance with Chinese national standard JGJ 55-2011 Specification for Mix Proportion Design of Ordinary Concrete [35]. Compaction was achieved using immersion vibrators to ensure uniform compactness. To compensate for the elevated water absorption characteristics of recycled aggregate concrete (RAC) during the initial 30 min post-mixing and ensure adequate workability, a supplementary water content was incorporated into the RAC specimen mixtures. This adjustment followed the experimental protocol established by Wu et al. [36]. After demolding, specimens were cured in a standard curing chamber (20 ± 2 °C, RH ≥ 95%) for 28 days. Prismatic specimens (100 × 100 × 400 mm), cubic specimens (100 × 100 × 100 mm), and test beams were subjected to identical curing regimes to maintain material property consistency. The fabrication of the stirrups and the curing of specimens are illustrated in Figure 6.

It should be particularly noted that the core of this study is to explore the performance degradation trends of recycled concrete beams under freeze–thaw environments. Given the long duration and high cost of long-term freeze–thaw salt erosion coupled tests, only one beam specimen was used under each working condition in this study. This was a strategic choice made under limited resources to prioritize obtaining the basic performance degradation trends over a wide range of freeze–thaw cycles. The load and strain data presented in the paper are all single-measurement values, primarily used to reveal the trending characteristics of performance degradation.

### 2.3. Freeze–Thaw Cycle Testing

#### 2.3.1. Freeze–Thaw Media

Freshwater environment: the control group utilized tap water to maintain a neutral pH.

Compound salt solution: following the literature [37], a composite salt solution (5% NaCl + 2% Na_2_SO_4_) was adopted to simulate the freeze–thaw conditions of the Lop Nur saline soil region. Rationale: the saline soil in Xinjiang’s Lop Nur region is classified as strong chlorine–saline soil, with soluble salts dominated by chlorides and supplemented by sulfates, and highly variable salt content [37]. Specifically: chloride ion (Cl^−^) content ranges from 2.86% to 4.06%, corresponding to NaCl concentrations of approximately 4.72–6.70%. The selected 5% NaCl concentration falls within this range and adequately represents typical local chloride levels. Sulfate ion (SO_4_^2−^) content varies from 1.44% to 14.62%, corresponding to Na_2_SO_4_ concentrations of 2.00–21.54%. The chosen 2% Na_2_SO_4_ concentration aligns with the lower bound of this range, balancing the need to reflect sulfates as a secondary component while avoiding excessive concentrations that could destabilize testing through rapid expansion.

The parameters (such as A, B, a, and b) in the subsequent models are all calibrated for the specific composite salt solution environment mentioned above. Therefore, when this study’s model is to be applied to freeze–thaw environments with different salt types and concentrations, the relevant parameters need to be experimentally redetermined based on the specific environment.

#### 2.3.2. Freeze–Thaw Treatment

Prior to freeze–thaw cycling, all specimens were immersed for 4 days in either tap water or the composite salt solution at 20 ± 2 °C. Freeze–thaw cycles were conducted using the rapid freeze–thaw method specified in the Standard for Test Methods of Long-Term Performance and Durability of Ordinary Concrete (GB/T 50082-2009) [38], employing a TR-TSDR-3 freeze–thaw testing machine. Specimens were treated via full immersion, with solution immersion depth maintained at 25 ± 5 mm. Each freeze–thaw cycle lasted approximately 4 h, with temperature ranging from +8 ± 2 °C to −17 ± 2 °C.

### 2.4. Static Loading Test Method

**Figure 7:**
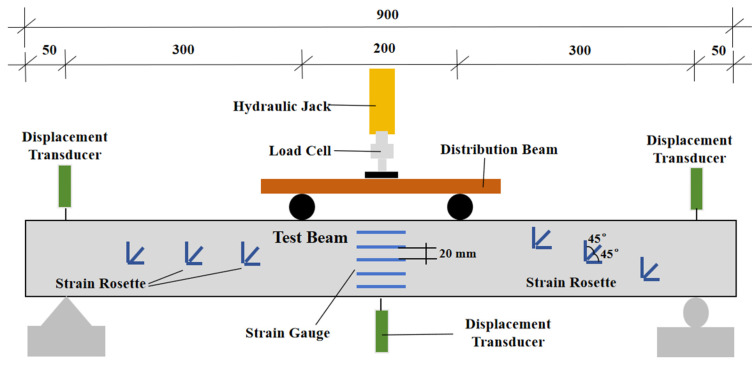
Measurement arrangement and load setup.

Static load testing was conducted subsequent to completion of the freeze-thaw cycling protocol. As illustrated in Figure 7, all specimens adopted a simply supported boundary condition. Displacement measurements were obtained through three linear variable differential transformers (LVDTs): one positioned at midspan and two located adjacent to the beam’s supports. For concrete strain monitoring, five electrical resistance strain gauges were vertically aligned at equidistant intervals across the depth of the midspan section, enabling quantification of strain distribution under incremental loading.

The experimental beams were subjected to a symmetrical two-point loading configuration. Loads were applied incrementally following a multi-stage protocol: testing commenced at an initial load level, with subsequent increments set at 10% of the predicted ultimate load. Upon reaching 90% of the predicted cracking load threshold, the load increment magnitude was reduced to 5% of the predicted ultimate load. Load measurements were recorded using a precision load cell, while a dynamic data acquisition system synchronized the collection of reinforcement strain, concrete surface strain, and displacement data at each load stage. Crack development was systematically monitored throughout the testing process using a digital crack width gauge, with continuous observation from initial load application until beam failure occurred.

For failure criteria, based on the specification [38], loading was terminated upon reaching any of the following ultimate state conditions: crushing failure of concrete in the compression zone. Fracture of the main longitudinal reinforcement (reinforcement strain ≥ 0.01) was as follows: maximum crack width ≥ 1.5 mm, spalling of concrete cover ≥ 30%, mid-span deflection ≥ L/50 (i.e., ≥ 18 mm).

## 3. Test Results and Discussion

### 3.1. Deterioration of Material Properties

#### 3.1.1. Mass Loss

**Figure 8:**
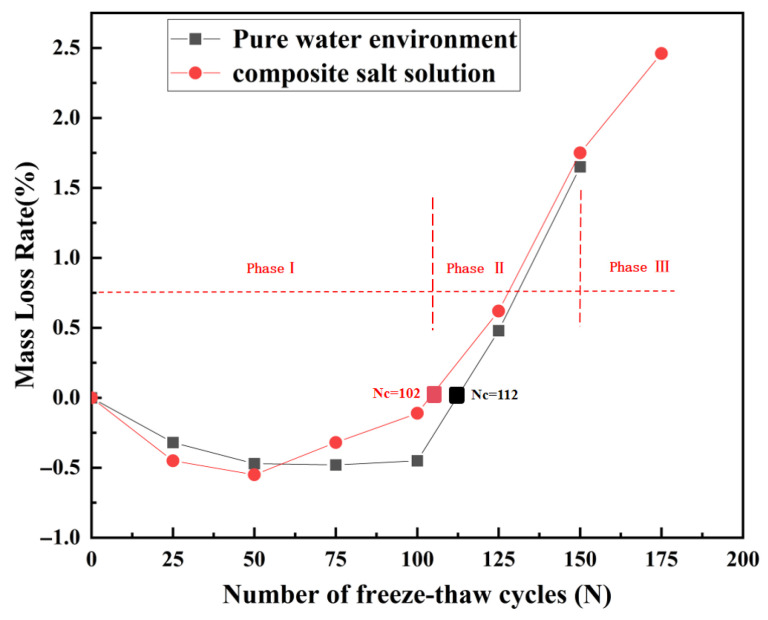
Evolution of mass loss for recycled aggregate concrete (RAC).

As shown in Figure 8, the mass variation of recycled aggregate concrete (RAC) under freshwater and composite salt solution environments exhibits a three-stage evolution during freeze–thaw cycles.

Phase I: Moisture absorption and expansion stage (with the number of cycles *N* ≤ 50). In both environments, the pattern of mass change in specimens is generally consistent with the freeze–thaw process of ordinary concrete [39]. Due to the temporary mass increase caused by solution absorption, the mass change rate during this stage is negative. However, the degree of deterioration is more pronounced in the composite salt environment: after 50 cycles, the mass loss rate reaches −0.55%, which is 0.08 percentage points higher than that in the freshwater environment (indicating a 17% faster rate of deterioration). This difference arises because, in the initial stage, salt ions mainly fill the pores and have not yet generated significant crystallization expansion or frost heave stress [40]. Meanwhile, the pore-refining effect of fly ash and the bridging effect of polypropylene fibers jointly suppress the development of damage [20], keeping the overall degree of damage at a relatively low level. The erosive effect of the compound salt environment becomes evident but remains within the material’s self-repair capacity.

Phase II: Dynamic equilibrium stage (50 < *N* ≤ 100 cycles): mass variation trends diverge between environments. In freshwater, the mass loss rate decreases to −0.33% due to C-S-H gel formation from fly ash secondary hydration, which repairs pores. Conversely, the compound salt environment shows a sharp increase in mass loss rate (3.1 times that of freshwater), attributed to accelerated Cl^−^ penetration through cracks and weakened interfacial transition zone (ITZ) bonding [41,42]. Here, salt erosion surpasses the material’s repair capacity, initiating accelerated damage accumulation.

Phase III: Accelerated spalling stage (*N* > 100 cycles): specimens in the compound salt environment reach mass loss equilibrium at 102 cycles, 10 cycles earlier than freshwater (112 cycles). By 175 cycles, the mass loss rate (2.46%) is 49% higher than freshwater. This disparity stems from synergistic erosion of compound salt ions dominating the damage process, leading to significantly faster deterioration compared to freshwater [43,44].

Notably, under identical conditions, the hybrid modification with fly ash and polypropylene fibers reduced mass loss rate to 0.62% after 125 cycles, far below the 5.70% reported for ordinary RAC [45]. This improvement is attributed to fly ash reducing salt solution permeability through pore refinement and polypropylene fibers constraining microcrack propagation via bridging effects. Experimental results validate the efficacy of this hybrid system in mitigating salt-freeze damage.

#### 3.1.2. Relative Dynamic Elastic Modulus (RDEM)

**Figure 9:**
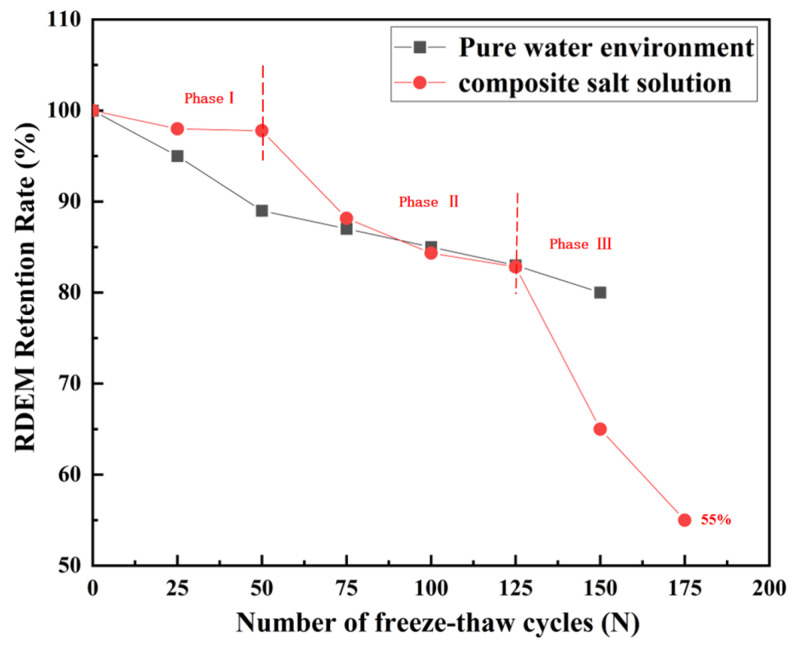
Relationship between RDEM retention rate and number of freeze–thaw cycles (N).

As shown in Figure 9, the relative dynamic elastic modulus (RDEM) retention rate of recycled aggregate concrete (RAC) exhibited a quasi-linear decline with increasing FT cycles (N) in both fresh water and composite salt solution environments.

Phase I: During the initial 50 cycles, RDEM retention in the compound salt environment stabilizes at 97%, with a minimal single-cycle decay rate of 0.04%. This slow decay is attributed to initial salt ion crystallization filling pores, while Cl^−^ and SO_4_^2−^ ions have not fully penetrated the concrete interior to induce significant chemical erosion or structural damage. The pore filling effect of salt ions temporarily suppresses early-stage damage, maintaining high structural integrity.

Phase II: During the freeze–thaw cycles, 50–125, RDEM retention in the compound salt environment undergoes a sharp decline, dropping to 82.23% at 125 cycles. This rapid decrease stems from Cl^−^’s concentration advantage and faster penetration rate, enabling preferential reaction with tricalcium aluminate (C_3_A) in cement to form Friedel’s salt, which temporarily inhibits massive AFt formation [44]. The filling effect of erosion products transiently enhances concrete compactness, keeping RDEM retention higher than in freshwater. Notably, compared to the 59.7% RDEM retention reported by Peng et al. [45] after 125 cycles, the modified RAC with hybrid fly ash and polypropylene fibers achieves 82.83%, demonstrating significantly improved freeze-thaw resistance. Although salt erosion begins to manifest, the inhibitory effect of the hybrid system remains dominant.

Phase III: During the freeze–thaw cycles, 125–175, RDEM retention in the compound salt environment accelerates, declining to 65% at 150 cycles and 55% at 175 cycles, i.e., 15 percentage points lower than freshwater at the same cycle counts, though still exceeding the 61.1% observed in 5% Na_2_SO_4_ single-salt environments [41]. This accelerated deterioration arises from two mechanisms: (1) Friedel’s salt decomposition under sustained SO_4_^2−^ exposure, leading to AFt formation and pore structure degradation, which propagates microcracks in the interfacial transition zone (ITZ) [38]; (2) salt solution lowering the pore water freezing point, prolonging liquid water freezing duration, thereby extending hydrostatic pressure cycles on pore walls and exacerbating crack expansion [41].

Overall, the decay characteristics of RAC’s RDEM retention are strongly environment-dependent: freshwater damage accumulates steadily with cycles, while compound salt environments induce staged deterioration due to Cl^−^-SO_4_^2−^ synergistic effects. The hybrid modification with fly ash and polypropylene fibers effectively delays this process, exhibiting superior freeze–thaw durability.

#### 3.1.3. Compressive Strength

**Figure 10:**
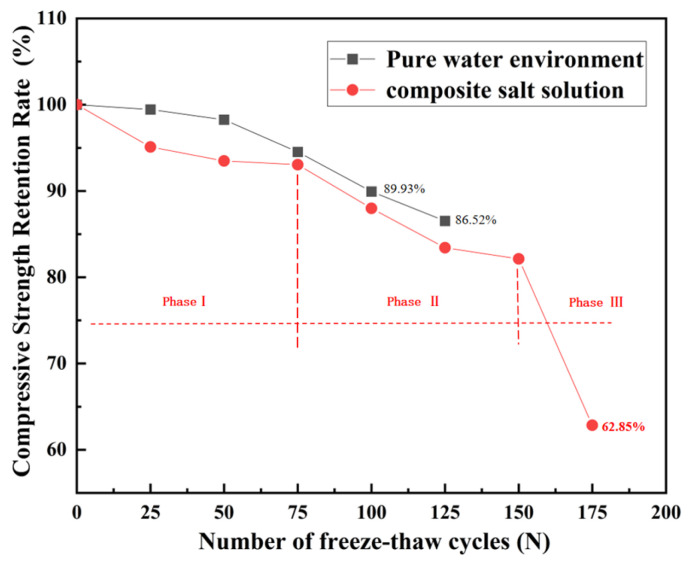
Relationship between compressive strength retention rate and freeze–thaw cycles (N).

As illustrated in Figure 10, the compressive strength of recycled aggregate concrete (RAC) in both freshwater and Cl^−^/SO_4_^2−^ compound salt environments exhibits a continuous decline with increasing FT cycles (N). Notably, the strength degradation in compound salt environments demonstrates a distinct three-stage characteristic.

(1) Phase I: Ion migration–frost heave dominant stage (0–75 cycles): Strength degradation in this stage is primarily dominated by frost heave effects induced by ion migration, with more severe damage observed in compound salt environments than in freshwater. After 75 cycles, the strength loss rate in compound salt reaches 6.95%, exceeding the freshwater environment by 1.48 percentage points (5.47%). This discrepancy arises because salt ion enrichment accelerates microcrack propagation in ITZ, expediting mechanical performance deterioration. Although sulfate ions temporarily mitigate pure physical frost heave, the combined effects of chemical erosion and synergistic ion interactions result in greater damage in compound salt environments compared to freshwater FT [46]. The synergistic interaction between ion migration and frost heave constitutes the primary degradation mechanism in this stage.
(2) Phase II: Chemical corrosion dominant stage (75–150 cycles): As cycles progress, chemical erosion gradually becomes the dominant factor in strength degradation, with accelerated strength loss observed in compound salt environments. After 100 and 150 cycles, strength loss rates reach 14.95% and 23.83%, respectively. Notably, at 125 cycles, the strength loss rate (13.48%) is significantly lower than the 51.6% reported by Peng et al. [45], indicating substantially improved degradation resistance. While chemical erosion dominates this stage, the inhibitory effect of the hybrid modification system continues to delay damage progression.
(3) Phase III: Structural failure stage (150–175 cycles): This stage witnesses a rapid escalation of structural damage culminating in failure, with strength loss rates exhibiting leapfrog growth. After 175 cycles, the strength loss rate surges to 34.18%, primarily due to preferential deterioration of initial defects in the ITZ and sustained degradation of bond performance, ultimately triggering macroscopic structural collapse [47].

In summary, the compressive strength degradation of RAC in compound salt environments progresses through three sequential stages: ion migration–frost heave, chemical corrosion, and structural failure. The hybrid modification system incorporating fly ash and polypropylene fibers effectively delays damage progression across all stages, significantly enhancing freeze–thaw resistance.

### 3.2. Compressive Strength Degradation Behavior of Recycled Aggregate Concrete

To quantitatively characterize the compressive strength degradation of RAC under compound salt solution freeze–thaw cycling, this study adopted an exponential decay model to establish the relationship between compressive strength $f_{c}(N)$ (MPa) and freeze–thaw cycle count (*N*). The model expression is as follows:

(1) $$f_{c}(N)=\alpha \times e^{bN}$$

where a is initial compressive strength (predicted strength at *N* = 0); b is decay coefficient.

Based on regression analysis of 0–175 freeze–thaw cycle test data, the fitted parameters were a = 35.04 MPa and b = −0.002433. The magnitude of b directly reflects the rate of freeze-thaw damage progression.

To validate model reliability, Equation (1) was used to predict compressive strengths at various freeze–thaw cycles, with predictions compared against experimental measurements (as detailed in Table 4).

Excluding the *N* = 0 discrepancy (+4.46%, attributed to curing inhomogeneity), all prediction errors remained below 3.0%. The model demonstrates excellent agreement with experimental observations (R^2^ = 0.983), confirming its validity for simulating freeze–thaw-induced strength degradation in composite salt environments.

### 3.3. Shear Behavior of Test Beams

#### 3.3.1. Crack Propagation and Failure Patterns

**Figure 11:**
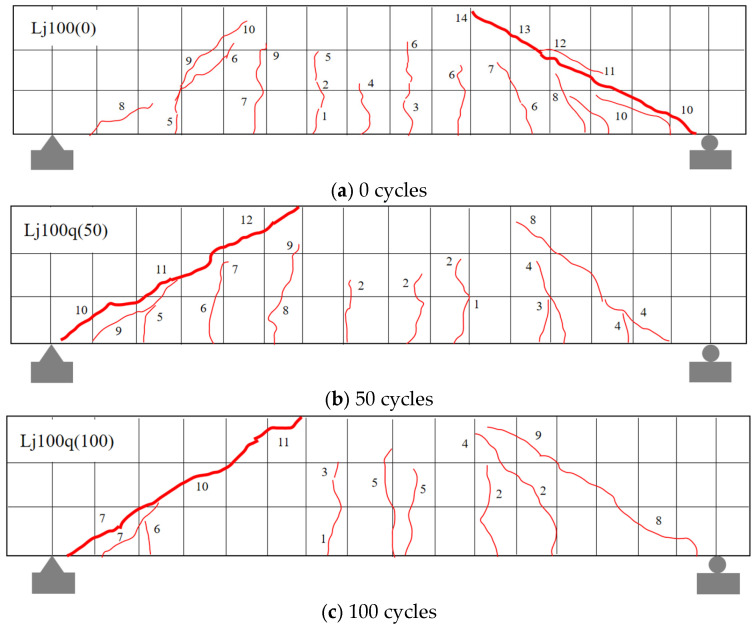
Crack failure patterns of recycled concrete beams in freshwater environment. Note: Numbers 1, 2, … indicate the sequence of crack initiation; red diagonal lines denote cracks, where thin lines represent secondary cracks and thick lines correspond to the primary cracks that ultimately lead to failure.

**Figure 12:**
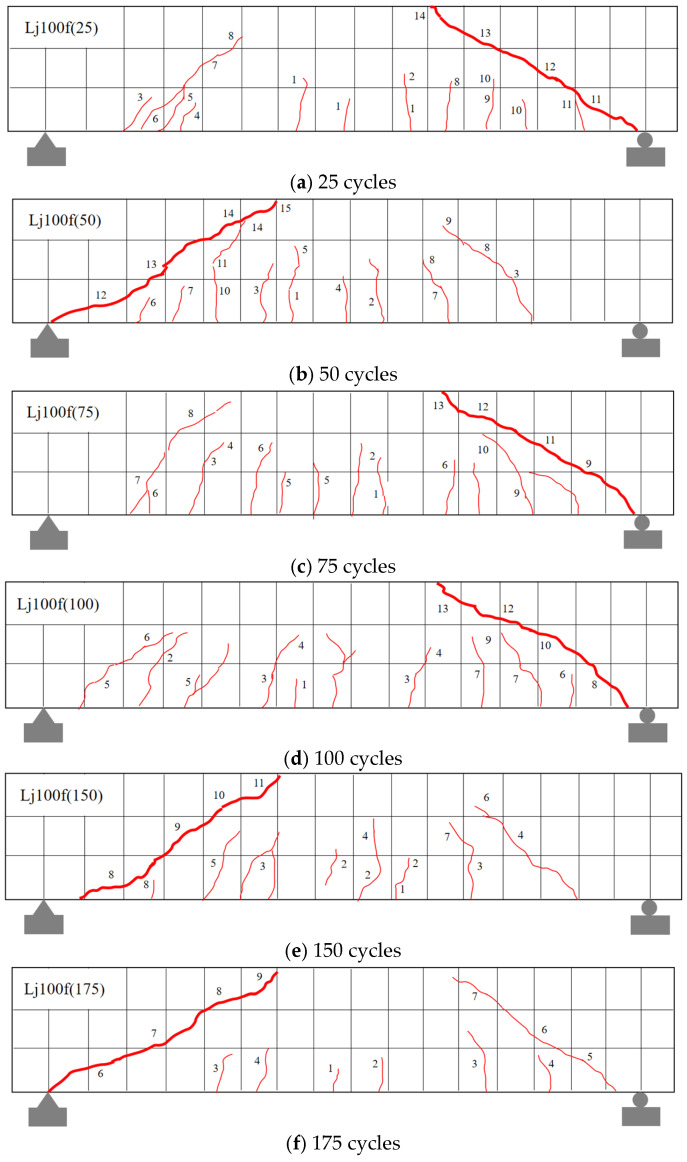
Crack failure patterns of recycled concrete beams in composite salt solution. Note: Numbers 1, 2, … indicate the sequence of crack initiation; red diagonal lines denote cracks, where thin lines represent secondary cracks and thick lines correspond to the primary cracks that ultimately lead to failure.

Based on crack observation results (Figure 11 and Figure 12), the recycled concrete beams subjected to freshwater and composite salt (Cl^−^/SO_4_^2−^) freeze–thaw cycles exhibited comparable failure modes, though differences existed in crack development characteristics.

Experimental results (Figure 11 and Figure 12) demonstrate that recycled aggregate concrete (RAC) beams under both freshwater and Cl^−^/SO_4_^2−^ compound salt freeze–thaw environments exhibit a typical shear compression failure mode: upon reaching critical load levels, vertical cracks initially appear in the flexural zone. As loading increases, the number of vertical cracks increases and their lengths extend. When the load reaches a certain magnitude, diagonal cracks emerge and propagate continuously in the shear zone, while mid-span vertical cracks stabilize. Ultimately, primary diagonal cracks in the shear zone coalesce, leading to diagonal shear failure. This failure pattern aligns with findings reported by Su et al. [9], Maruyama et al. [48], and Arezoumandi et al. [49], confirming the universality of shear-compression failure modes in RAC beams.

**Figure 13:**
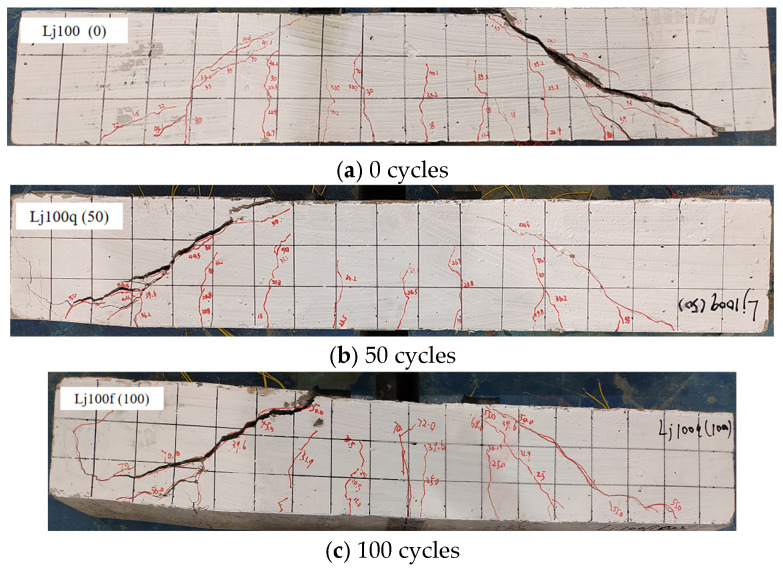
Appearance of RAC beam specimens after failure under non-freeze–thaw and freshwater freeze–thaw conditions. The numbers on the test specimen represent the trajectory of the load and cracks during the experimental loading process.

**Figure 14:**
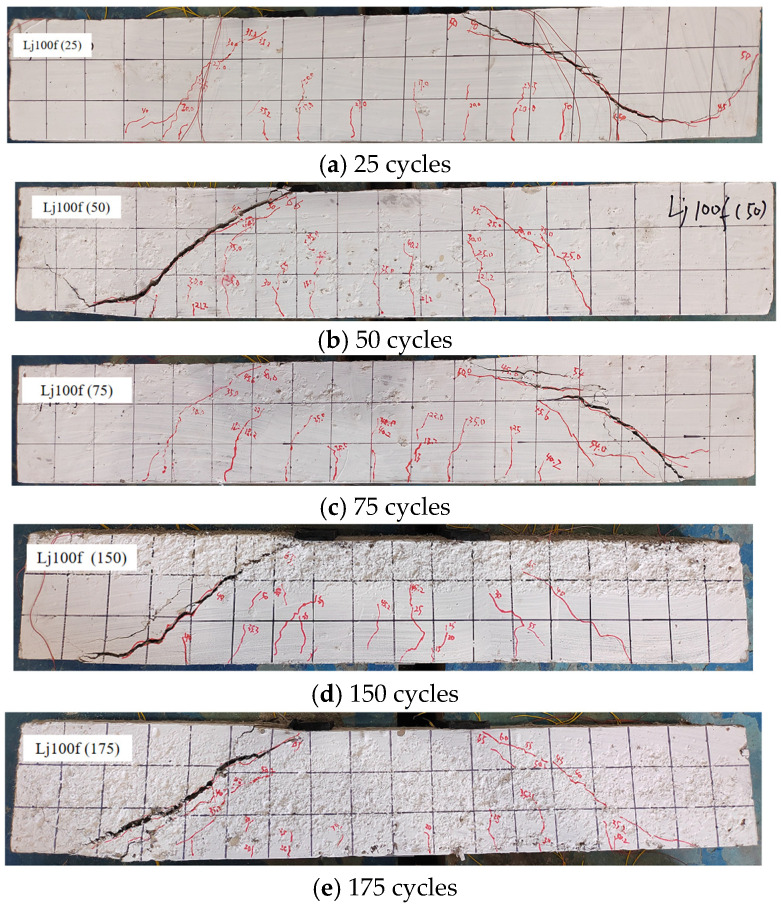
Appearance of RAC beam specimens after failure under in composite salt solution. The numbers on the test specimen represent the trajectory of the load and cracks during the experimental loading process.

From the appearance of the failed test beams (Figure 13 and Figure 14), two types of key characteristics can be clearly observed. First, there are flexural failure characteristics, specifically manifested as vertical flexural cracks in the mid-span region and their complete propagation process. Second, there are shear failure characteristics, allowing for a direct view of the morphological details and extension directions of the diagonal cracks in the shear-affected zones. It is particularly noteworthy that the effect of polypropylene fibers can be distinctly observed at the cracks. These fibers effectively tie the concrete fragments together and significantly inhibit the rapid propagation of cracks. This phenomenon serves as direct evidence that the specimens possess superior ductility.

Beyond crack characteristics, the concrete strain distribution was further analyzed to clarify section deformation behavior.

#### 3.3.2. Concrete Strain Distribution in Test Beams

**Figure 15:**
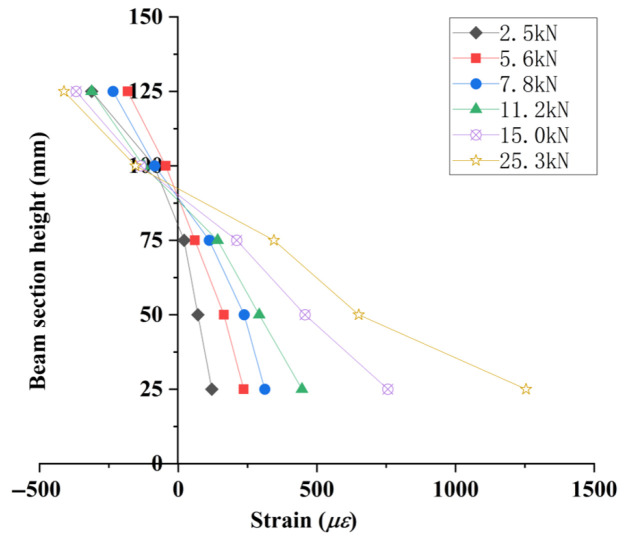
Concrete strain distribution curves (unfrozen state).

**Figure 16:**
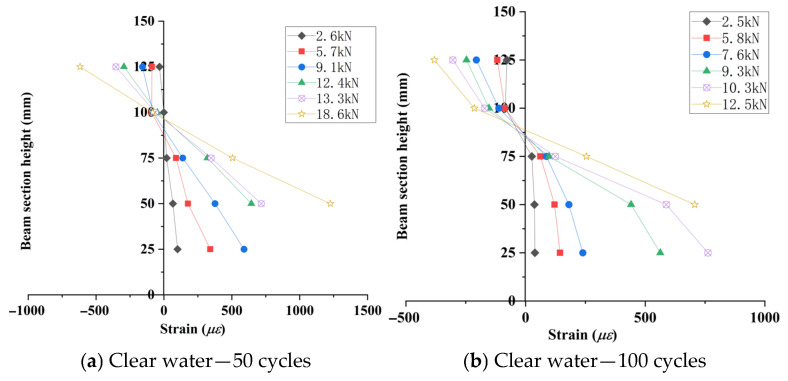
Concrete strain distribution curves in clear water environment.

**Figure 17:**
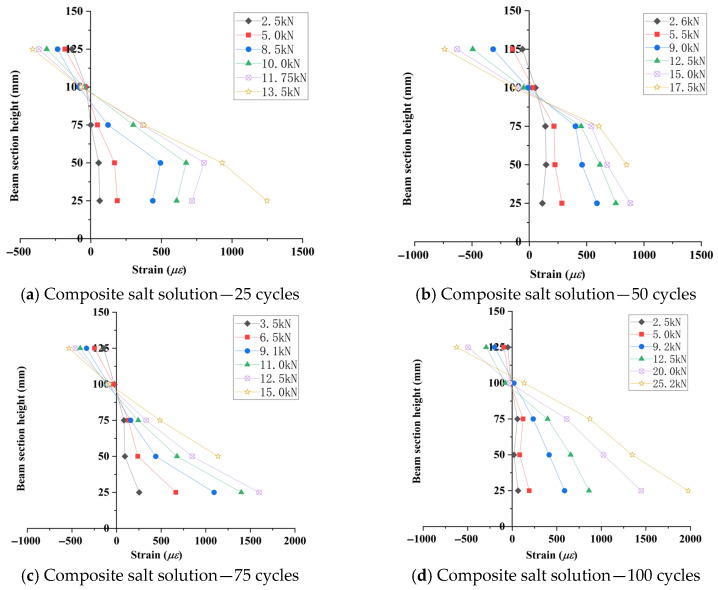

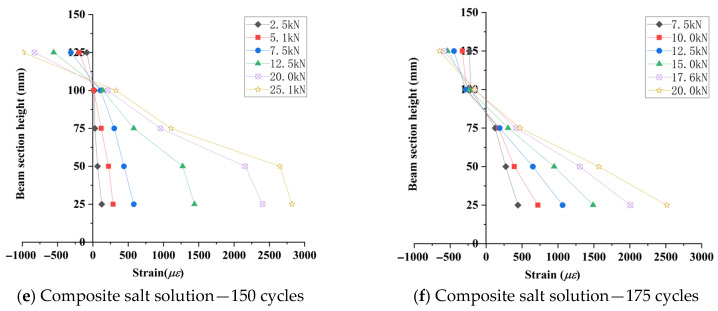
Concrete strain distribution curves in a composite salt solution environment.

Five strain gauges were equidistantly mounted at the mid-span section of the beam flexural zone (Figure 7). Sectional strain data collected during incremental loading were used to generate strain distribution profiles along the beam depth (Figure 15, Figure 16 and Figure 17).

**Figure 18:**
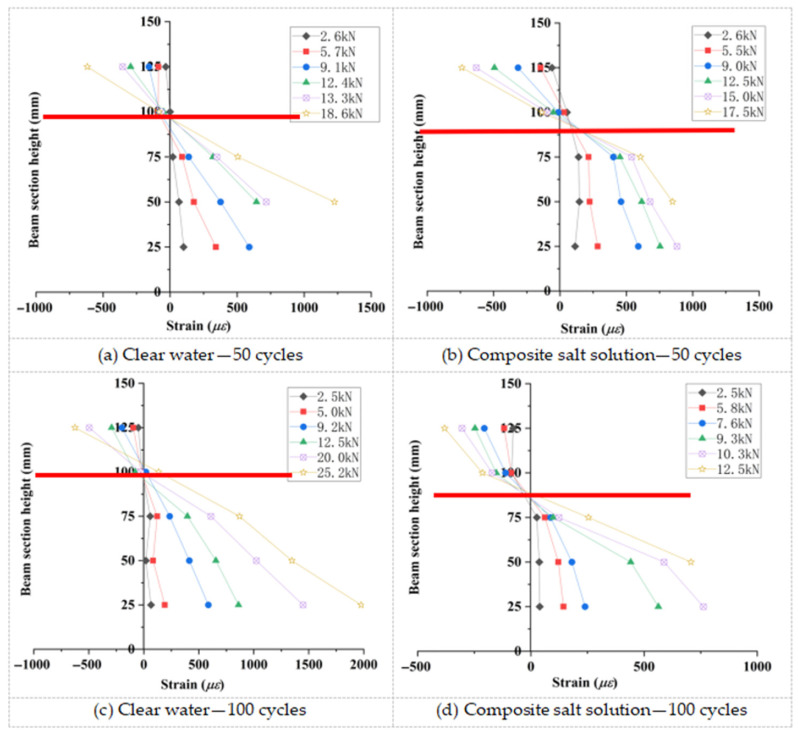
Comparative strain profiles along mid-span section depth. Note: the red lines represents the position of the neutral axis.

As shown in Figure 15, Figure 16, Figure 17 and Figure 18, the strain distribution across the mid-span section of RAC beams under unfrozen (0 cycles), freshwater, and compound salt freeze–thaw conditions all conform to the plane section assumption, indicating that freeze-thaw damage does not alter the overall deformation compatibility of the section.

Figure 18 reveals that after 50 and 100 freeze–thaw cycles, the compressive strength loss rates in compound salt environments are 2.8 times and 1.5 times those in freshwater environments, respectively. This phenomenon arises from the lower compressive strength of RAC, which causes a downward shift of the neutral axis, aligning with findings reported by Su (2019) [9].

#### 3.3.3. Load–Stirrup Strain Analysis of Test Beams

The yield strain of stirrups was calculated using Equation (2) based on the relationship between steel yield strength and elastic modulus:

ε = *f*_y_/*E*_s_ = 467 × 10/(2.1 × 10^5^) = 2224 με (2)

where *f*_y_ is stirrup yield strength (MPa); *E*_s_ is stirrup elastic modulus (MPa).

The calculated yield strain threshold is defined as 2224 με. The measured maximum strain values of stirrups in test beams under different freeze–thaw environments (in με) are presented in Table 5.

**Figure 19:**
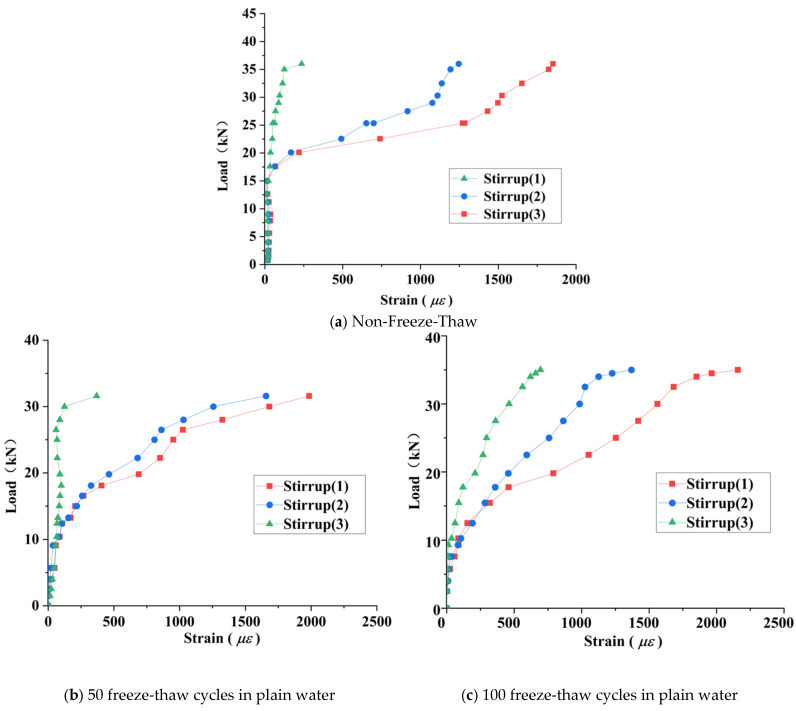
Load–stirrup strain curves in freshwater environment.

Figure 19 presents the load–strain curves of stirrups in RAC beams after varying freeze–thaw cycles (note: some strain gauges failed during freeze–thaw processes). Table 5 summarizes the maximum stirrup strain values. The results indicate that prior to diagonal crack initiation, strain development in stirrups remained minimal across all specimens. Following diagonal crack formation, stirrups intersecting the cracks (e.g., stirrups #2 and #3 in Figure 19a) exhibited significant strain increases, while non-intersecting stirrups (e.g., stirrup #1 in Figure 19b) continued to develop strain gradually. This demonstrates that stirrups contribute minimally to shear transfer before beam cracking, with shear resistance primarily activating post-cracking, a finding consistent with Su (2019) [9]. The underlying mechanism relates to the inherent lower tensile strength of RAC due to RCA internal microcracks, with freeze–thaw damage further accelerating deterioration and inducing premature shear compression failure prior to stirrup yielding.

**Figure 20:**
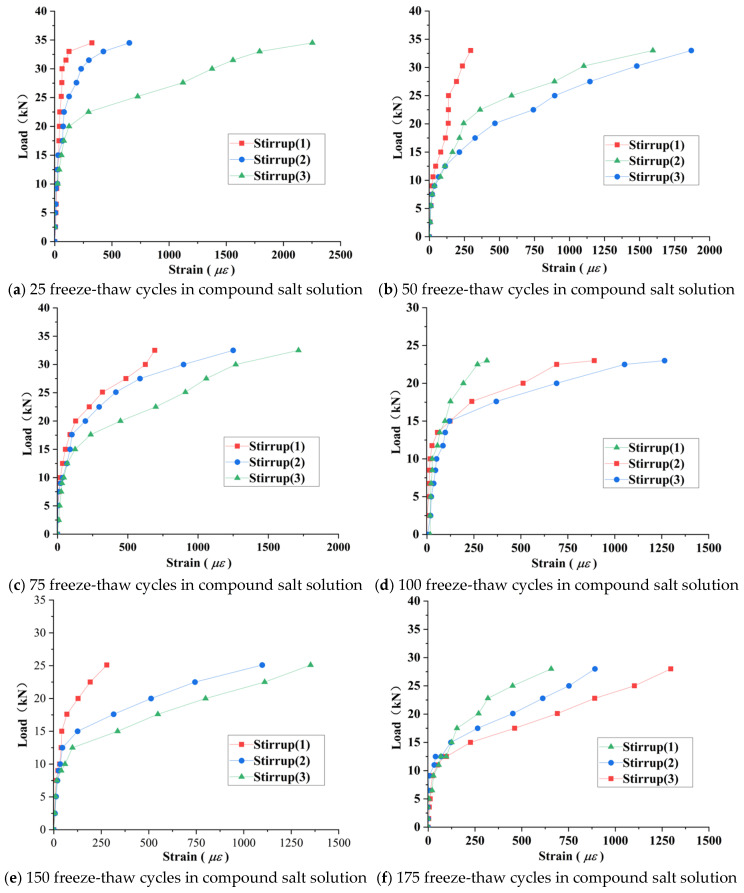
Load–stirrup strain curves in a composite salt environment.

As shown in Figure 20, in the compound salt solution environment after 50 freeze–thaw cycles, microcrack propagation and bond degradation occurred, leading to increased stirrup load sharing ratio. At 100 cycles or more, interface bond deterioration weakened stirrup confinement but specimens remained unfailed. At 175 cycles, strain growth rate accelerated (peak strain not captured due to strain gauge failure), with compressive strength loss reaching 34.2%. Additionally, as freeze–thaw cycles increased and the failure mode transitioned from ductile to brittle, the erosion–frost heave synergistic effect in compound salt solutions accelerated interface degradation. Under equivalent freeze–thaw cycles, stirrup strain increments in compound salt environments exceed those in freshwater environments.

#### 3.3.4. Load–Deflection Response of the Test Beams

Table 6 presents the measured normal section cracking load (*F*_n_) and ultimate load (*F*_u_). The critical inclined crack initiation load, as defined in this study, corresponds to the test load applied via the hydraulic jack (Figure 19) that generated the maximum observable crack length. Beyond this load level, further loading increments caused crack widening without additional propagation. Ultimate load capacity is defined as the test load at which shear-induced failure occurred.

Table 6 demonstrates that in both freshwater environment and compound salt solution, the normal section cracking load of RAC beams decreases with increasing FT cycles. After 100 freeze–thaw cycles in freshwater and compound salt solution, the normal section cracking loads of RAC beams with 100% recycled coarse aggregate (RCA100%) decreased to 30.68% and 35.51% of the unfrozen beams, respectively. The shear capacity of RAC beams continuously declined with freeze–thaw cycles, showing reductions of 12.22% and 22.22% compared to unfrozen beams after 100 cycles in the respective environments. The phenomenon of reduced cracking load and ultimate bearing capacity in frozen-thawed RAC beams has also been confirmed by Su et al. [9]. The primary mechanisms include the following: (1) degradation of concrete tensile strength with increased freeze–thaw cycles, and (2) impaired bond performance between steel reinforcement and concrete due to freeze–thaw damage. Furthermore, RAC beams exposed to compound salt solution exhibited lower shear capacity than those in freshwater after equivalent freeze–thaw cycles. This discrepancy arises from the synergistic effects of chloride and sulfate ions in the salt solution, which induce significant crystalline stress and expansive products within the recycled coarse aggregates (RCA). These processes promote internal crack initiation and propagation, while crack expansion further accelerates the accumulation of crystalline stress and expansive products, ultimately leading to faster attenuation of concrete’s tensile and shear strengths.

**Figure 21:**
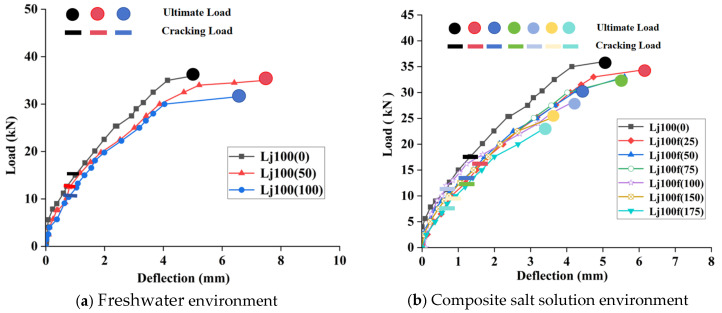
Load–deflection curves of RAC beams under different freeze–thaw conditions.

Figure 21a presents the load–deflection curves of RAC beams subjected to 0, 50, and 100 freeze–thaw cycles in a freshwater environment. The curves exhibited consistent overall trends: approximately linear behavior under low loads with minimal deflection increase, indicating elastic working stage of the beams; as loading progressed, the curve slope gradually decreased, reflecting continuous stiffness degradation; ultimate failure occurs due to complete stiffness loss, with longitudinal reinforcement remaining unyielded at failure. This phenomenon is attributed to the reduction in the RCA elastic modulus and deformation resistance caused by freeze–thaw actions in freshwater.

Figure 21b shows the–load deflection curves of RAC beams after 0–175 freeze–thaw cycles in compound salt solution. The general pattern aligns with freshwater environment: linear load–deflection relationship at initial loading stage with limited deflection growth. With increasing load, the curve slope continuously decreased, indicating progressive stiffness attenuation, leading to failure due to insufficient bearing capacity. Comparative analysis revealed that under equivalent freeze–thaw cycles, RAC beams in salt solution exhibited significantly lower stiffness and weaker deformation resistance compared to freshwater environment, demonstrating the enhanced erosiveness and damage potential of salt solution. This finding corroborates studies by Wang et al. [49] and Arezoumandi et al. [50], which concluded that Cl^−^ and SO_4_^2−^ ions in salt solution synergistically accelerate stiffness degradation: Cl^−^ reduces the freezing point of pore solutions to prolong freeze–thaw cycles, while SO_4_^2−^ reacts with hydration products to induce microcrack propagation, collectively exacerbating beam deterioration.

### 3.4. Shear Capacity Prediction Model for Recycled Aggregate Concrete Beams

#### 3.4.1. Necessity of Modifying the Shear Capacity Model for Post-Freeze–Thaw RAC Beams

In the initial research design, we indeed noted that directly applying model parameters established for NAC to predict the performance of RAC might lead to systematic deviations. This aligns with the prevailing international research findings. As cited in this paper, studies by Tošić et al. (2019) [51] and Zamanzadeh et al. (2015) [52] both pointed out that due to the failure to account for key characteristics of recycled aggregates, such as elastic modulus, shrinkage, creep, and interfacial synergistic effects, existing models significantly underestimate the long-term deformation or shear capacity of recycled concrete members. Therefore, in predicting the shear capacity of the inclined section of RAC simply supported beams under a composite salt freeze–thaw environment, without specialized parameter calibration tailored to the properties of recycled aggregates, existing models indeed struggle to ensure predictive accuracy, making their modification necessary.

To verify the aforementioned viewpoint, the experimental shear capacities of RAC beams were compared with the predicted results from three existing models, including those proposed by Zhang et al. [19], Su et al. [9], and the Chinese code DG/TJ 08-2018-2007 [53].

Zhang et al.’s model [19] established an analytical expression for calculating the shear capacity of RAC beams as follows:

(3) $$F_{u}=0.88\times 0.175\times \frac{f_{c}bh_{0}}{1+\lambda }+1.07\times \frac{h_{0}f_{yv}A_{sv}}{s}$$

where $f_{c}$ is the axiressive s compstrength of RAC; λ is the shear–span ratio; $h_{0}$ is the effective height of the beam; $f_{yv}$ is the tensile strength of the stirrups; *A*_sv_ is the cross-sectional area of the stirrup; and s is the stirrup spacing.

Su al.’s model [9] proposed a predictive framework for RAC beam shear capacity, defined by

(4) $$F_{u}=\eta (N)\times (0.88\times 0.175\times \frac{f_{c}bh_{0}}{1+\lambda }+1.07\times \frac{h_{0}f_{yv}A_{sv}}{s})$$

$$\eta (N)=7\times 10^{-6}N^{2}-0.0028N+0.985(N>0)$$

where N is the number of freezing-and-thawing cycles of the RAC beams.

The Chinese technical specification DG/TJ 08-2018-2007 [53] provides the following formula for RAC beam shear capacity calculation:

(5) $$F_{u}=0.85\times (0.7\times f_{t}bh_{0}+1.25\times \frac{h_{0}f_{yv}A_{sv}}{s})$$

Comparative analysis between experimental results and model predictions is presented in Figure 20. The post-freeze–thaw evaluation revealed that the predictive models developed by Zhang et al.’s model [19], Su et al.’s model [9], and DG/TJ 08-2018-2007 [53] consistently overestimated the residual shear capacity of RAC beams after freeze–thaw exposure.

**Figure 22:**
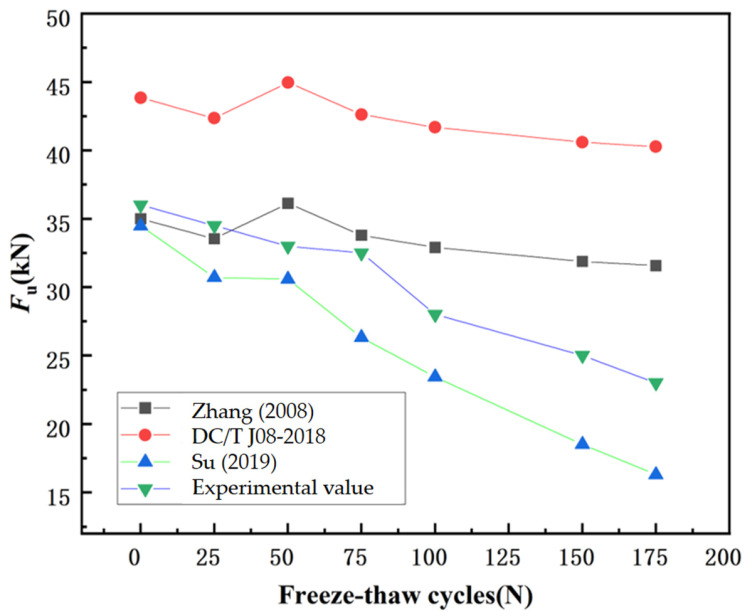
Comparative analysis of experimental shear capacity values versus predicted values from selected models and local standards [9,19].

Figure 22 shows that significant deviations exist when using Zhang et al.’s model [19], Su al.’s model [9], and the Technical Specification for Application of Recycled Aggregate Concrete (local standard) [53] to predict the shear capacity of recycled aggregate concrete (RAC) beams after compound salt freeze-thaw cycles. Zhang et al.’s model [19] showed relatively close agreement with experimental values before 75 freeze–thaw cycles, but the relative error reached 9.46% and expanded to 37.26% as freeze–thaw cycles increased. The prediction accuracy of other models also failed to meet requirements. Therefore, existing models need modification to accurately predict the shear capacity of inclined sections in simply supported beams under compound salt freeze–thaw conditions.

#### 3.4.2. Modification of Shear Capacity Prediction Model for Post-Freeze–Thaw RAC Beams

According to the Code for Design of Concrete Structures (GB 50010-2010) [33], the shear capacity of inclined sections in beams is jointly provided by concrete (*P*_c_) and stirrups (*P*_s_). However, existing research models exhibit significant limitations.

Most studies utilize a global reduction factor K to characterize freeze–thaw effects. For instance, Yan et al. [54] introduced a conversion coefficient k related to recycled coarse aggregate (RCA) replacement ratio r and freeze–thaw cycles N, but failed to differentiate their distinct degradation patterns. Some investigations, such as Zhang [50], only focus on concrete strength degradation while neglecting indirect effects from freeze–thaw-induced interfacial bond deterioration.

Additionally, studies by Deng et al. [4], and Tabsh and Abdelfatah [5] demonstrate that 100% RCA concrete undergoes more severe strength degradation than ordinary concrete under compound salt freeze–thaw cycles, necessitating special consideration in models.

To address this, a comprehensive influence coefficient *k*_v_(N) is introduced to account for both concrete tensile strength degradation and interfacial bond deterioration. The modified shear capacity formula is expressed as

(6) $$F_{uN}=k_{v}(N)V_{uj,N}=k_{v}(N)\times (F_{\mathrm{cN}}+F_{\mathrm{sN}})$$

where *F*_u,N_ denotes the modified shear capacity of inclined sections for polypropylene fiber-reinforced concrete beams after N FT cycles;

*F*_cN_ and *P*_sN_ represent the shear contributions from concrete and reinforcement after N cycles, respectively;

and *k*_v_(N) correlates with concrete tensile strength degradation and interfacial bond deterioration. Concrete degradation exhibits cumulative effects with increasing FT cycles.

Abderrahmane Rhardane et al. (2020) [55] indicated that such degradation follows an exponential law, thus the attenuation model is assumed as

(7) $$\begin{array}{c} \alpha_{c}(N)=\exp(aN)\;\;\;\;\;\;\alpha_{b}(N)=\exp(bN)\\ \ldots \end{array}$$

where *a*_c_(N) and *a*_b_(N) represent the concrete degradation coefficient and interfacial bond deterioration coefficient, respectively; a and b are negative decay rate parameters, and N denotes freeze–thaw cycles.

The coefficient *k*_v_(N) integrates both concrete degradation and interfacial bond damage effects. Considering their differential contributions to shear capacity degradation, its expression is defined as

(8) $$k_{v}(N)=A\times \alpha_{c}(N)+B\times \alpha_{b}(N)$$

where A and B are influence weights for concrete degradation and interfacial bond damage, respectively, satisfying A + B = 1. A reflects the dominant role of concrete, while B accounts for the secondary effect of interfacial bond deterioration.

According to Jie Liu et al. [56], concrete contributes 66.2–100% to shear capacity within stirrup reinforcement ratios of 0–0.5%, positively correlated with concrete strength. With a stirrup ratio of 0.28% in this study, the concrete contribution ratio approximates 0.80, aligning with the “concrete-dominated shear degradation” pattern confirmed by Aimin Yuan et al. [57].

Given the 100% RCA usage in this experiment, concrete strength degradation in compound salt freeze–thaw environments is more severe than in ordinary concrete, while stirrup–concrete interfacial bond strength deteriorates relatively slowly. Therefore, the concrete degradation weight for RAC beams should exceed 0.8. Based on the literature [58,59,60,61,62,63], weights are set as A = 0.95 (concrete degradation) and B = 0.05 (bond damage) in freshwater, versus A = 0.85 and B = 0.15 in compound salt solution. Through the regression analysis of experimental data, decay parameters are determined as a = −0.003201, b = −0.014399 for freshwater; and a = −0.001668, b = −0.06926 for compound salt solution. The modified shear capacity prediction models are presented in Equations (9) and (10):

Freshwater environment:

(9) $$F_{uN}=F_{uj,N}[0.95\times \exp(-0.003201N)+0.05\times \exp(-0.014399N)]$$

Compound salt solution: (10)

(10) $$F_{uN}=F_{uj,N}[0.85\times \exp(-0.001668N)+0.15\times \exp(-0.06926N)]$$

#### 3.4.3. Validation of the Modified Models

Based on Equations (9) and (10), the shear capacity of inclined sections in beams subjected to compound salt freeze–thaw cycles was predicted and compared with experimental values, as shown in Table 7 and Table 8.

Table 6 reveals that under freshwater conditions (50–100 cycles), the modified model (Equation (9)) achieves a mean predicted/measured ratio of 1.00 with a standard deviation of 0.000 and coefficient of variation 0.00%. This indicates nearly identical agreement between predictions and experiments, demonstrating the model’s high accuracy and practical applicability.

Table 8 demonstrates that under compound salt solution conditions (25–175 cycles), the modified model (Equation (10)) yields a mean ratio of 1.0583 with a standard deviation of 0.0204 and coefficient of variation 0.0193%. The close alignment between predicted and measured values confirms the model’s high precision, structural stability, and strong practicality.

#### 3.4.4. Validation of Model Applicability

To verify the applicability of the modified prediction model, it was applied to predict experimental data from Su et al.’s study [9], with the results presented in Table 9.

As shown in Table 8, under 25–130 freeze–thaw cycles, the mean ratio of predicted-to-experimental values reaches 1.048, closely approaching the theoretical value of 1.0, indicating high prediction accuracy under freeze–thaw conditions. The standard deviation of 0.0507 suggests moderate data dispersion, while the coefficient of variation (4.84%) demonstrates relatively low relative variability. These metrics collectively confirm the model’s stable performance and strong practicality across different freeze–thaw cycles.

It should be particularly noted that this study constructs a shear capacity prediction model based on the experimental data obtained from a 100% replacement rate of RCA. The model parameters A, B, a, and b are all calibrated using this experimental data. If the model is to be applied to other RCA replacement rates, the coefficients A, B, a, and b must be recalibrated through experiments.

## 4. Conclusions

This study experimentally investigated the shear performance degradation of polypropylene fiber-reinforced recycled aggregate concrete beams under FT cycles in composite salt solution. A shear capacity prediction model was established, with main conclusions as follows:

(1) In both freshwater and composite salt solution environments, the crack patterns and distribution characteristics of freeze–thaw-damaged PF-RAC beams demonstrated consistent behavior: initial microcracks consistently initiated within the ITZ under tension, with principal cracks propagating along approximately 45° shear paths. The crack bridging effect of PPF effectively restricted disorderly crack propagation, thereby mitigating environmental impacts on crack development. This finding aligns with the conclusions of Yap et al. (2013) [64] regarding the role of fibers in maintaining structural integrity under harsh conditions, confirming the universal applicability of fiber-mediated crack control.
(2) Compressive strength continuously deteriorated with increasing FT cycles in both environments, yet the degradation rate was significantly higher in the composite salt solution. For instance, after 125 cycles, the strength loss reached 22.11% in salt environment versus 13.48% in freshwater; after 175 cycles, the loss increased to 34.18%. This accelerated deterioration is attributed to the synergistic effect of salt crystallization pressure and chemical erosion, as also described by Valenza and Scherer (2007) [65]. Importantly, the incorporation of PPF substantially alleviated strength loss. After 125 cycles, PF-RAC exhibited a loss of only 13.48%, far lower than the 51.6% reported by Peng et al. (2022) [45] for plain RAC under similar conditions, quantitatively confirming that fibers enhance frost resistance through matrix toughening and microcrack restraint.
(3) Both cracking load and shear capacity decreased with freeze–thaw cycles, with more severe degradation in the composite salt environment. After 100 cycles, the shear capacity reduction was 22.22% in salt solution, about 1.70 times that in freshwater. After 175 cycles, the cracking load dropped from 17.6 kN to 7.6 kN in salt solution, compared to 12.2 kN after only 100 cycles in freshwater. These results confirm that Cl^−^ and SO_4_^2−^ ions act synergistically to accelerate stirrup corrosion and concrete matrix damage, significantly impairing residual load capacity. However, it should be noted that the present study employed a fixed PPF content, length, and diameter. The potential existence of a fiber effectiveness threshold under combined salt freeze–thaw conditions, as well as the limitations of fiber reinforcement, remain unexamined and warrant further investigation.
(4) The modified shear capacity model incorporating the comprehensive influence coefficient kv(n) demonstrated high predictive accuracy. In freshwater, errors were below 1%; in composite salt environment, errors remained under 8%. When validated using external data from Su et al., errors stayed below 9%, confirming the model’s broad applicability. This model offers a practical tool for quantifying the coupled effect of freeze–thaw and salt erosion on the shear behavior of fiber-reinforced recycled aggregate concrete members, addressing a gap in current design guidelines.

This study is limited by the single-specimen design, and thus fails to report data variability indicators (such as standard deviations). Future research should increase the number of specimens under each working condition (three or more parallel specimens) to provide more reliable statistical references.

In this study, the type and dosage of PPF, as well as the freeze–thaw cycling regime, were set as singular variables, which imposes certain limitations on the research scope. Future research should focus on the following directions:

Firstly, it is necessary to investigate the impact of different fiber types (such as steel fibers and basalt fibers), fiber dosages, and fiber blending schemes. This will enable a comprehensive evaluation of the regulatory effects of fiber parameters on the salt–freeze resistance performance of FR-RAC beams.

Secondly, microscopic testing techniques including SEM (Scanning Electron Microscope), XRD (X-ray Diffraction), and MIP (Mercury Intrusion Porosimetry) can be utilized to dynamically track the damage evolution patterns of materials during salt freeze–thaw cycles. Based on this, a multi-scale analysis model encompassing the “ITZ—Concrete Matrix—Beam Structure” can be constructed. This model will quantitatively reveal the intrinsic connections between microscopic damage and macroscopic mechanical properties, providing theoretical support for the durability design of FR-RAC beams.

Thirdly, key design parameters such as effective beam height (h_0_), stirrup reinforcement ratio (ρ_s_), and RCA replacement rate (r) need to be incorporated. This will further refine and establish a comprehensive prediction model for shear performance that covers multiple influencing factors, enhancing the model’s adaptability to real-world engineering scenarios.

Furthermore, a finite element model will be constructed to conduct reverse verification and parametric expansion of the experimental results in this study. This will provide a more solid theoretical foundation for the generalized application of the proposed model, and broaden its scope of applicability.

## Figures and Tables

**Table 1:** Key performance indicators of raw materials.

Material Name	Fundamental Properties
Ordinary Portland Cement (P·O 42.5)	Density: 3.10 g/cm^3^, specific surface area: 349 m^2^/kg, loss on ignition (LOI): 3.57%, residue on 80 μm sieve: <10%, chemical composition (wt.s%): CaO 58.62; SiO_2_ 23.08; Al_2_O_3_ 6.01.
Fly Ash (Grade I, FA)	Loss on ignition (LOI): 2.62%; fineness (45 μm sieve residue): 16%; Al_2_O_3_ content: 36.8%; SiO_2_ content: <45.1%; SO_3_ content: 1.2%; chloride ion content: 0.015%; specific surface area: 420 m^2^/kg.
Polypropylene Fiber (PPF)	Monofilament polypropylene fibers produced by Shandong Jinhongyao Engineering Materials Co., Ltd. (Jinan City, China) were used, with a diameter of approximately 36 μm, a density of about 0.91 g/cm^3^, a length of approximately 12 mm, a tensile strength > 430 MPa, an elongation > 35%, a breaking strength of 455 MPa, an initial modulus of 4200 MPa, and resistance to acids and alkalis.
Mixing Water	No specific performance indicators (meets general tap water quality requirements for concrete mixing).
Additives (FK-AE + DFTR-PCE)	Complies with Technical Code for Application of Concrete Admixtures (GB 50119-2013) [29].

**Table 2:** Characteristic parameters of particle size distributions for fly ash and cement.

Material Type	D10 (μm)	D50 (μm)	D90 (μm)
Fly ash	2.859	11.92	30.62
Cement	2.978	14.36	46.97

**Table 3:** Mix design of recycled freeze-resistant concrete.

Specimen Type	Cement (kg/m^3^)	FA (%)	PPF (%)	RCA (kg/m^3^)	Sand (kg/m^3^)	Water (kg/m^3^)	Water Reducer (kg/m^3^)
Test Beam/ Prism/Cube	418.44	20	0.9	1085	535	185	5.29

**Table 4:** Comparison of measured and predicted compressive strengths.

Freeze–Thaw Cycle (N)	0	25	50	75	100	125	150	175
Measured Strength (MPa)	33.65	33.06	32.00	30.31	28.62	26.21	25.63	22.15
Predicted Strength (MPa)	35.04	32.97	31.02	29.19	27.47	25.85	24.32	22.89
Relative Error (%)	4.46	0.36	2.12	2.44	2.48	0.52	2.92	1.58

**Table 5:** Measured maximum strain values of stirrups in the test beams (με).

Environmental Condition	Unfrozen	Clear Water		Composite Salt Solution					
Freeze–Thaw Cycles	0	50	100	25	50	75	100	150	175
Maximum Strain, με	1852	2158	1985	2219	1875	1780	1310	1374	1308

**Table 6:** Shear performance degradation data of test beams under different environments (units: Fn/Fu in kN; crack width/deflection in mm).

Environmental Condition	Specimen ID	Freeze–Thaw Cycles	*F*_n_, kN	*F*_u_, kN	Maximum Inclined Crack Width, mm	Maximum Midspan Deflection, mm
Clear water environment	Lj100(0)	0	17.6	36	1.15	5.05
	Lj100q(50)	50	15.4	35	2.46	7.46
	Lj100q(100)	100	12.2	31.6	1.59	6.59
Composite salt solution environment	Lj100f(25)	25	14.8	34.5	1.23	6.23
	Lj100f(50)	50	13.5	33	1.60	5.60
	Lj100f(75)	75	11.35	32.5	0.41	5.41
	Lj100f(100)	100	10.60	28	0.13	4.13
	Lj100f(150)	150	8.85	25	1.59	3.59
	Lj100f(175)	175	7.60	23	0.36	3.36

Notes: (1) Lj100q(x): freshwater-exposed specimens subjected to x freeze–thaw cycles; Lj100f(x): composite salt solution-exposed specimens subjected to x freeze–thaw cycles, “100” indicates 100% recycled coarse aggregate (RCA) replacement ratio by volume.

**Table 7:** Comparison between predicted and measured values using modified models (fresh water environment).

Freezing and Thawing Cycles	*F*_ue_, kN	[48] *F*_up_, kN	Equation (9) *F*_up_, kN	*F*_ue_/*F*_up_
0	36	38.48	38.48	0.94
50	35	38.37	35.00	1.00
100	31.6	35.10	31.62	1.00

Note: *F*_ue_ denotes experimental shear capacity values; *F*_up_ represents predicted shear capacity values derived from the modified model.

**Table 8:** Comparison between predicted and measured values using modified models (compound salt solution).

Freezing and Thawing Cycles	*F*_ue_, kN	[33] *F*_up_, kN	Equation (10) *F*_up_, kN	*F*_ue_/*F*_up_
0	36	38.48	38.48	0.94
25	34.5	37.22	32.33	1.07
50	33	39.41	31.00	1.06
75	30.5	37.44	28.11	1.08
100	28	36.67	26.39	1.06
150	25	35.76	23.67	1.06
175	23	35.48	22.52	1.02

Note: *F*_ue_ denotes experimental shear capacity values; *F*_up_ represents predicted shear capacity values derived from the modified model.

**Table 9:** Validation of the modified model applicability.

Shear–Span Ratio	Cross-Sectional Dimensions (mm × mm)	Freeze–Thaw Cycles	RCA Replacement Rate (%)	*F*_ue_, kN	Equation (9) *F*_up_, kN	*F*_ue_/*F*_up_
2.48	100 × 150	0	100	72.67	74.49	0.98
2.48	100 × 150	25	100	64.94	66.9	0.97
2.48	100 × 150	55	100	61.84	60.25	1.03
2.48	100 × 150	80	100	58.75	55.23	1.06
2.48	100 × 150	105	100	57.20	52.18	1.10
2.48	100 × 150	130	100	52.57	48.48	1.08

Note: *F*_ue_ denotes experimental shear capacity values; *F*_up_ represents predicted shear capacity values derived from the modified model.

## Data Availability

The original contributions presented in this study are included in the article. Further inquiries can be directed to the corresponding author.

## Abbreviations

The following abbreviations are used in this manuscript:

RCA	Recycled coarse aggregate
RAC	Recycled aggregate concrete
NAC	Natural aggregate concrete
ITZ	Interfacial transition zone
FT	Freeze–thaw
PPF	Polypropylene fibers
FA	Fly ash
RDEM	Relative dynamic elastic modulus
AFt	Ettringite
PF-RAC	Polypropylene fiber-reinforced recycled aggregate concrete
C-S-H	Calcium silicate hydrate
LVDT	Linear variable differential transformer
UNEP	The United Nations Environment Programme
ACI	American Concrete Institute
DG/TJ	Shanghai Local Technical Specification
GB	Guobiao (Chinese National Standard)
XJJ	Xinjiang Local Standard
RH	Relative humidity
